# Experimental and Theoretical Design on the Development of Matrix Tablets with Multiple Drug Loadings Aimed at Optimizing Antidiabetic Medication

**DOI:** 10.3390/pharmaceutics16121595

**Published:** 2024-12-14

**Authors:** Mousa Sha’at, Lacramioara Ochiuz, Cristina Marcela Rusu, Maricel Agop, Alexandra Barsan (Bujor), Monica Stamate Cretan, Mihaela Hartan, Adrian Florin Spac

**Affiliations:** 1 Department of Pharmaceutical Technology, Faculty of Pharmacy, “Grigore T. Popa” University of Medicine and Pharmacy, 16 Universității Street, 700115 Iasi, Romania; mousa-shaat@umfiasi.ro (M.S.); monica.stamate@umfiasi.ro (M.S.C.); 2 Department of Physics, “Gheorghe Asachi” Technical University of Iasi, Prof. Dr. Docent Dimitrie Mangeron Rd., No. 59A, 700050 Iasi, Romania; cristina-marcela.rusu@academic.tuiasi.ro (C.M.R.); magop@tuiasi.ro (M.A.); 3 Romanian Scientists Academy, 54 Splaiul Independentei, 050094 Bucharest, Romania; 4 Independent Researcher, 57 Canta Street, 700530 Iasi, Romania; mihaela_hartan@yahoo.com; 5 Department of Physico-Chemistry, Faculty of Pharmacy, “Grigore T. Popa” University of Medicine and Pharmacy, 16 Universității Street, 700115 Iasi, Romania; adi_spac@yahoo.com

**Keywords:** matrix tablets, metformin hydrochloride, honokiol, mathematical models, multifractal dynamics, multiple drug, dissolution test

## Abstract

**Background:** Diabetes is a growing global health crisis that requires effective therapeutic strategies to optimize treatment outcomes. This study aims to address this challenge by developing and characterizing extended-release polymeric matrix tablets containing metformin hydrochloride (M-HCl), a first-line treatment for type 2 diabetes, and honokiol (HNK), a bioactive compound with potential therapeutic benefits. The objective is to enhance glycemic control and overall therapeutic outcomes through an innovative dual-drug delivery system. **Methods:** The tablets were formulated using hydrophilic polymers, such as Carbopol^®^ 71G NF and Noveon^®^ AA-1. The release kinetics of M-HCl and HNK were investigated through advanced mathematical models, including fractal and multifractal dynamics, to capture the non-linear and time-dependent release processes. Traditional kinetic models (zero-order, first-order, Higuchi equations) were also evaluated for comparison. In vitro dissolution studies were conducted to determine the release profiles of the active ingredients under varying polymer concentrations. **Results:** The study revealed distinct release profiles for the two active ingredients. M-HCl exhibited a rapid release phase, with 80% of the drug released within 4–7 h depending on polymer concentration. In contrast, HNK demonstrated a slower release profile, achieving 80% release after 9–10 h, indicating a greater sensitivity to polymer concentration. At shorter intervals, drug release followed classical kinetic models, while multifractal dynamics dominated at longer intervals. Higher polymer concentrations resulted in slower drug release rates due to the formation of a gel-like structure upon hydration, which hindered drug diffusion. The mechanical properties and stability of the matrix tablets confirmed their suitability for extended-release applications. Mathematical modeling validated the experimental findings and provided insights into the structural and time-dependent factors influencing drug release. **Conclusions:** This study successfully developed dual-drug extended-release matrix tablets containing metformin hydrochloride and honokiol, highlighting the potential of hydrophilic polymers to regulate drug release. The findings emphasize the utility of advanced mathematical models for predicting release kinetics and underscore the potential of these formulations to improve patient compliance and therapeutic outcomes in diabetes management.

## 1. Introduction

Diabetes is one of the fastest-growing medical conditions and represents a health emergency of the 21st century [1]. The global prevalence of diabetes in 2021 was 10.5%, with an estimated increase to 12.2% by 2045 [1]. Given the projected rise in the number of individuals affected, there is an urgent need not only to develop new therapeutic approaches but also to enhance the efficacy of existing antidiabetic drugs. Addressing this need is crucial to effectively managing the growing burden of the disease. This objective can be achieved either by synthesizing innovative molecules or by optimizing and improving the therapeutic profiles of currently available treatments through modified drug delivery systems [2,3]. The formulation and optimization of polymeric matrix antidiabetic tablets with multiple drug loadings represent an important innovation in diabetes therapy, offering significant advantages in controlling the release of active substances and improving patient compliance with treatment [4,5]. The use of polymeric matrices enables controlled and prolonged release of active substances, thereby reducing fluctuations in plasma concentrations and improving glycemic control [6]. Honokiol (HNK), or 5,3′-diallyl-2,4′-dihydroxybiphenyl (C_18_H_18_O_2_, Mr = 266.3 g/mol), is derived from the bark of Magnolia officinalis (dried bark from the stem and branch, containing a minimum of 2% honokiol and magnolol) and the flower of Magnolia officinalis (steamed and dried unopened flower, containing a minimum of 0.2% honokiol and magnolol) [7]. From a health perspective, HNK is a promising therapeutic agent for various conditions, including diabetes mellitus, diabetic peripheral neuropathy, cancer, neurodegenerative diseases, nonalcoholic fatty liver disease, reproductive disorders, and arthritis [8,9,10].

Metformin hydrochloride (M-HCl), also known as N,N-dimethylimidodicarbonimidic diamide or 1,1-dimethylbiguanide hydrochloride (C_4_H_12_ClN_5_, Mr = 165.62 g/mol), is a small molecule used as a first-line therapy in the pharmacological treatment of diabetes mellitus, particularly type 2. It improves insulin sensitivity, reduces glycemic levels, and inhibits gluconeogenesis in the liver [11]. Due to these properties, metformin (as a senotherapeutic agent) has garnered significant interest from the scientific community and pharmaceutical companies for its potential applications in human anti-aging treatments [12]. Both active molecules can be quantitatively analyzed using High-Performance Liquid Chromatography (HPLC), mass spectrometry, or UV spectroscopy [11,12,13,14,15,16,17].

Noveon and Carbopol have been used successfully in commercial formulations for decades and are highly efficient gel-forming and matrix table binders, allowing for controlled drug release tablets, taste masking, and mucoadhesion. The choice of Carbopol 71G NF is based on the fact that it is suitable for direct compression; it is a prolonged release excipient, an effective diluent, and suitable for solid oral dosage forms. The unique feature of the Carbopol 71G NF polymer is its free-flowing granular form, allowing for tablet manufacturing by direct compression. However, in order to achieve a robust final dosage form, a direct compression formulation requires an excipient like Carbopol 71G NF that exhibits good flowability and compressibility. Other advantages of Carbopol and Noveon include the following: optimal performance as extended release polymers or controlled release polymers; ability to produce smaller, easier to swallow tablets compared to other polymers like cellulosics; highly efficient gel matrix formation, used either alone or with other co-excipients, which are effective at low polymer usage levels; ability to produce multimedia compliant, controlled release oral solid dose formulations in combination with a broad spectrum of active pharmaceutical ingredients (APIs) and co-excipients; excellent mucoadhesion properties and taste masking of bitter drugs and versatile processing options, such as direct compression [18].

The aim of this study is to develop matrix tablets with modified release based on the acrylic derivatives Carbopol 71G NF and Noveon AA-1, using metformin hydrochloride and honokiol as active pharmaceutical ingredients. These molecules are formulated into polymeric matrix tablets with modified release properties, representing innovative formulations designed for antidiabetic therapy with multiple drug loadings. The matrix tablets were prepared through direct compression, utilizing the two matrix-forming agents in different concentrations: Carbopol 71G NF at 15% and 25%, and Noveon AA-1 at 3% and 7%. Based on the analysis of the dissolution test results, the study aims to develop mathematical models that predict the release kinetics of the two active molecules, metformin hydrochloride and honokiol, in relation to physicochemical phenomena.

## 2. Materials and Methods

### 2.1. Chemicals and Reagents

Metformin hydrochloride or 1,1-Dimethylbiguanide hydrochloride (M-HCl, 97% purity, C_4_H_11_N_5_·HCl; Mr = 165.6) was obtained from Sigma Aldrich Chemie GmbH (Steinheim, Germany), honokiol (HNK, 98% purity, C_18_H_18_O_2_; Mr = 266.3) was obtained from New Natural Biotechnology (Shanghai, China), sodium acetate or sodium ethanoate (CH_3_COONa, ≥99%; Mr = 82.03) was purchased from Chimreactiv SRL (Bucharest, Romania), potassium dihydrogen phosphate (KH_2_PO_4_, ≥99.5%; Mr = 136.01) was purchased from Utchim SRL (Ramnicu Valcea, Romania), potassium chloride (KCl, ≥99%; Mr = 74.5) was purchased from Silal Trading SRL (Bucharest, Romania), acetic acid, glacial (CH_3_COOH, 99.9%; Mr = 60.1) was obtained from Chemical Company S.A. (Iasi, Romania), hydrochloric acid, concentrated (HCl, ≥37%; Mr = 36.46) was purchased from Chimreactiv SRL (Bucharest, Romania), sodium hydroxide (NaOH, 98.5%; Mr = 40.00) was obtained from Chemical Company S.A. (Iasi, Romania), and methanol (CH_3_OH, Mr = 32.04) for HPLC, ≥99.9% (Chromasolv™) was purchased from Honeywell Riedel-de Haën (Seelze, Germany). Ultrapure water (resistivity of 18.2 MΩ·cm, TOC < 10 ppb and bacterial count < 10 CFU/mL) was obtained from a local pharmaceutical company. Distilled water was obtained with the equipment of distillation GFL type 2004, no. 11918315J (Burgweld, Germany). All reagents used meet the quality requirements in accordance with national and European standards and are used as such, without any additional purification operations. Carbopol^®^ 71G NF Polymer (carbomer homopolymer type A or carbomer homopolymer) and Noveon^®^ AA-1 (Noveon^®^ Polycarbophil) were obtained from The Lubrizol Corporation (Wickliffe, OH, USA). Magnesium stearate (C_36_H_70_MgO_4_, 4.0–5.0% of magnesium content; Mr = 591.24) was obtained from Union Derivan S.A (Barcelona, Spain). MicroceLac^®^ 100 is a co-processed excipient obtained from Meggle Group (Wasserburg, Germany). The multicomponent matrix tablets (M-HCl/HNK) are based on extended-release polymers, using the granular Carbopol^®^ 71G and Noveon^®^ AA-1.

### 2.2. Equipments

The chromatography equipment used was an Agilent Technologies 1200 (Santa Clara, CA, USA) liquid chromatograph equipped with a multidiode detector (DAD type G1315B), quaternary pump (type G1311A), degasser (type G1322A), thermostatted column compartment (type G1316A) and Agilent ChemStation 32 software (Rev. B.03.02.). The chromatographic column used was a Thermoscientific ODS Hypersyl^TM^ (250 mm length, 4.6 mm inner diameter and 5 μm particle size), Vilnius, Lithuania. The pH determination was carried out using pH-meter inoLAB pH 7110 (Xylem Analytics Germany GmbH, Weilheim, Germany), PH CHECK G 5040-0302 (article 6311940, Dostmann electronic GmbH, Wertheim, Germany). Analytical balance PIONEER^®^ Analytical OHAUS PX124M (Ohaus Corporation, Parsippany, NJ, USA), ultrasonic bath Biobase (model UC-40A, Biobase Biodustry, Shandong, Co., Ltd., Jinan, China), water bath Biobase (model SY-1L4H, Biobase Biodustry, Shandong, Co., Ltd., Jinan, China), dissolution apparatus SR 8 Plus Dissolution Test Station (model 73-100-104, Hanson Research, Chatsworth, CA, USA), microliter™ Syringes, 20 μL Hamilton Bonaduz AG (CH-7402 Bonaduz, Switzerland), Rotilabo^®^—Mikroliterpipette 0.5–5.0 mL (article TA 26.1, Carl Roth GmbH, Karlsruhe, Germany), Pipet4u^®^ Pro 20–200 μL (article OK99957, AHN^®^ Biotechnologie GmbH, Nordhausen, Germany), Transferpette^®^ Dig. 100–1000 μL (article 704180, Brand GmbH + CO KG, Wertheim, Germany), and Korsch EK0 tablet press (12 mm flat punches, 5 kN tableting pressure) (Korsch AG, Berlin, Germany) were used.

### 2.3. Development of Polymeric Matrix Tablets with Multiple Drug Loading

Two powder mixtures were formulated and prepared, using the following active substances: metformin hydrochloride (400 mg/tablet) and honokiol (100 mg/tablet). For the matrix-forming excipients, Carbopol 71^®^ 71G NF and Noveon^®^ AA-1 were used in two concentrations: 15% and 25%, and 3% and 7%, respectively. Magnesium stearate was used at a concentration of 0.7% in all formulations, in accordance with the general monograph for tablets—“Compressi. Tabulettae” of the 10th edition of the Romanian Pharmacopoeia, which allows a maximum of 1%, being included for its lubricant role [18]. In each formulation, MicroceLac^®^ 100 was used as a diluent agent up to a final mass of 800 mg/tablet. This pharmaceutical excipient contains 75% α-lactose monohydrate and 25% microcrystalline cellulose, making it ideal for solid oral pharmaceutical forms. The tablets were obtained by direct compression using a Korsch EK0 tablet press. This method of tablet production is frequently used today, especially with the advancement of directly compressible excipients, as the manufacturing process is simpler, involves fewer steps, is cost-effective, and has high productivity, making it the first choice for pharmaceutical technologists.

### 2.4. Dissolution Studies

In vitro dissolution tests were performed according to the specifications of the “2.9.3. Dissolution Test for Solid Pharmaceutical Forms” and “5.17. Recommendations on methods for dosage forms testing” of the European Pharmacopoeia, 11th edition [7].

The dissolution tests were performed using the paddle apparatus (apparatus no. 2), keeping the temperature constant at 37 ± 0.5 °C throughout the test, regardless of the pH of the dissolution medium. For in vitro dissolution studies, the following dissolution media were prepared: for simulated gastric fluid with pH = 1.2 (0.37 g KCl, 0.75 mL concentrated HCl and distilled water up to 100 g) and simulated intestinal fluid pH = 6.8 (0.68 g KH_2_PO_4_, 2.24 mL 1 M NaOH solution and distilled water up to 100 g). After obtaining the gastric and intestinal simulated dissolution media, the pH of the solutions was determined and checked with the pH-meter, and subsequently adjusted with concentrated HCl or 1 M NaOH solution if necessary. The multi-component tablets (M-HCl and HNK) with prolonged release were placed at the bottom of the hemispherical-bottomed cylindrical vessel in the dissolving medium so that no air bubbles are present at the tablet-dissolving medium interface. The apparatus was switched on, the paddle rotation speed was selected and the dissolution test was performed for the 2 samples analyzed (noted P-1 and P-2). The dissolution test for the extended-release tablets was performed in two dissolution media with different pH (1.2 and 6.8), which simulated gastric and intestinal media. First, pH = 1.2 (gastric simulated medium) was used for the first 2 h; 60 rpm with constant temperature maintained at 37 ± 0.5 °C, after which the medium was replaced with an intestinal simulated medium, pH = 6.8 for 10 h, under the conditions of agitation and temperature. In total, 2 mL of sample was collected at different time intervals: 0.5, 1, 2, 3, 4, 5, 6, 7, 8, 9, 10, 11 and 12 h for prolonged-release tablets. Following each sample, the same volume of brand-new dissolving medium was added to the cylindrical jar at 37 °C to maintain a constant volume. At the designated period, samples were taken from the distance between the paddle and the dissolving medium’s surface, but also at least 10 mm from the vessel’s wall. They were then filtered using nylon filters with a 0.45 μm. The samples were analyzed using the HPLC method and validated in house using Agilent Technologies 1200 (USA). 

An Agilent Technologies 1200 (USA) liquid chromatograph with a thermostatted column compartment (type G1316A), quaternary pump (type G1311A), multidiode detector (DAD type G1315B), degasser (type G1322A), and Agilent ChemStation 32 software (Rev. B.03.02) was used to perform the chromatographic separation of HNK and M-HCl on a Thermoscientific ODS Hypersyl^TM^ column (250 mm × 4.6 mm, 5 μm) kept at 35 °C. The mobile phase used was 0.02 M acetate buffer (pH = 3)/methanol (15/85, *v*/*v*) as the mobile phase, with a flow rate of 1 mL/min. The detection wavelengths were 236 nm for M-HCl and 256 nm for HNK, and the injected volume was 20 µL. The method was validated according to the ICH Q2 (R1) and ICH Q2 (R2) guidelines [19,20]. Following the validation of the method, it proved that the method is linear in the range of 4–1200 μg/mL for M-HCl and 1–300 μg/mL for HNK. The regression equations obtained were *P_A_* = 90.3852 × *C* + 901.5302 for M-HCl (r^2^ = 0.9992) and *P_A_* = 51.1980 × *C* + 67.5673 for HNK (r^2^ = 0.9998), where *P_A_* is peak area and *C* is concentration in μg/mL. The method shows good precision (RSD < 2% for both HNK and M-HCl, in intra- and inter-day studies) and accuracy (mean recovery = 100.09% in the range 99.80–100.43% for M-HCl and mean recovery = 100.12% in the range 99.70–100.38% for HNK). The identification of the peaks corresponding to HNK and M-HCl was carried out by comparing the retention times of the peaks in the chromatogram of the samples with those obtained for standard solutions containing HNK (Rt = 3.935 ± 0.197 min) and M-HCl (5.722 ± 0.286 min), as well as of the absorption spectra of the two compounds with the standard spectra obtained under the same conditions. The quantitative analysis was performed based on the area of the peaks corresponding to HNK and M-HCl depending on the concentration used for calculating the equations of the calibration curves obtained after validation. The stability of the solution derived from the dissociation studies was also studied. Thus, the solutions taken from dissolution medium consisting of simulated gastric fluid pH = 1.2 and simulated intestinal fluid pH = 6.8 were analyzed immediately after sampling and after 24 h after sampling and no significant differences were observed between them, which demonstrates the fact that the solutions are stable for at least 24 h so that the method of quantitative determination of HNK and M-HCl can be used in dissolution studies.

A dilution factor (*DF*) was added to the calculation equation when the solution was diluted twice and reanalyzed if the area of the peak corresponding to M-HCl and HNK was larger than that found for standard M-HCl (800 μg/mL) and standard HNK (200 μg/mL). Six samples (tablets) were subjected to the dissolution test utilizing the SR 8 Plus Dissolution Test Station (model 73-100-104, Hanson Research, Chatsworth, LA, USA).

The amount of M-HCl and HNK released from the prolonged-release tablets was calculated using Equations (1)–(3):

(1) $$C_{1}\%=DF\cdot \frac{P_{A(tx)}-Int}{S}\cdot \frac{500}{1000}\cdot \frac{100}{A}$$

(2) $$C_{2}\%=DF\cdot \frac{P_{A\left(tx-1\right)}-Int}{S}\cdot \frac{500}{1000}\cdot \frac{2}{500}\cdot \frac{100}{A}$$

(3) $$C\%=C_{1}\%+C_{2}\%$$

where:

*C%* = percentage release in the dissolution medium;
*C*_1_*%* = percentage concentration calculated for the first sampling;
*C*_2_*%* = percentage concentration calculated in the 2 mL taken previously;
*DF* = dilution factor (1 or 2);
*P_A_* = peak area (mAU·min);
*Int* and *S* = intercept and slope of the regression line, respectively;
*A* = declared content (mg);
*tx* = current sampling time;
*tx* − 1 = previous sampling time.

### 2.5. Statistical Analysis

Data of the whole experiment were expressed as mean ± SD (n=3). The non-parametric Kruskal–Wallis test, implemented on Matlab and Statistics Toolbox Release 2020a (The MathWorks, Inc., Natick, MA, USA) was used to test the statistical significance of differences between the corresponding formulations P-1 and P-2. The post hoc test was employed to test the significance of the differences in the means. In our analysis, the level of significance is 0.05, so a difference with a *p*-value *p* < 0.05 was considered significant. The Kruskal–Wallis test (also called the one-way ANOVA on ranks) is a non-parametric version of the one-way ANOVA test, which is used to compare the means of two or more populations when the hypotheses for the ANOVA test (such as the normality or the homoscedasticity of data) are not satisfied. The Kruskal–Wallis test does not make any prior assumptions on the distribution of data. 

### 2.6. Theoretical Design

Developing a mathematical model capable of explaining and predicting the evolution of previously mentioned systems in the context of classical physics (based on the idea of continuity and differentiability of its physical quantities) is a difficult task due to the interdependence of phenomena. Models proposed in the classical sense, such as zero order, first order, Higuchi, Hixson–Crowell, Korsmeyer–Peppas, Baker–Lonsdale, Weibull, Hopfenberg, Gompertz and regression models [20,21,22], are empirical models, which are not based on phenomenological concepts, swinging only their validity by accepting approximations, even sometimes forced. In these conditions, there was a natural need to develop mathematical models that provide predictability of release kinetics in relation to physicochemical phenomena. As an alternative, fractal/multifractal analysis proved to be a useful tool in characterizing such systems.

## 3. Results and Discussion

### 3.1. In Vitro Dissolution Studies

**Figure 1:**
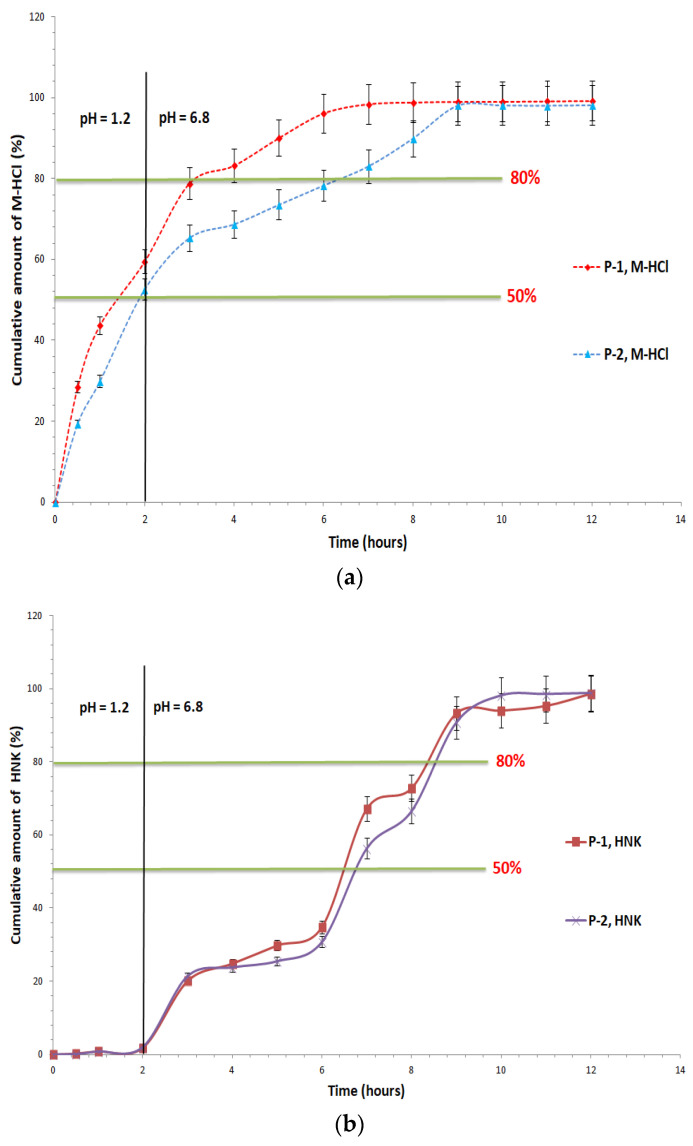
In vitro dissolution profile of (**a**) M-HCl and (**b**) HNK from tested prolonged-release tablets.

The in vitro dissolution studies conducted on hydrophilic matrix tablets revealed a prolonged release profile for both metformin hydrochloride and honokiol, closely dependent on the concentration of the matrix-forming polymers. By avoiding ‘dose dumping’, it was observed that the first sampling of prolonged-release tablets containing 400 mg of M-HCl and 100 mg of HNK in simulated gastric fluid (pH = 1.2) showed a release of M-HCl of 28.42% (M-HCl) and 0.32% (HNK) for the P-1 sample, and 19.33% (M-HCl) and 0.13% (HNK) for the P-2 sample. These results are in accordance with the acceptance criteria of the European Pharmacopoeia, 11th edition [7]. Formulation P-1 shows a maximum release of 59.50% (M-HCl), 1.86% (HNK), 52.62% (M-HCl) and 2.01% (HNK) in P-2 after two hours in the simulated stomach fluid with pH = 1.2 (Figure 1).

The second specification point for the dissolution test occurs when about half of the active compound is released from prolonged-release tablets. In simulated gastric fluid with pH = 1.2, this point is reached for M-HCl after two hours. For HNK, the second point is reached in 7 h in simulated intestinal fluid with a pH of 6.8 for both formulations (P-1—67.17% and P-2—56.28%). The last requirement in the guidelines of European Pharmacopoeia, 11th edition, is the guarantee of full release, with a minimum of 80% attainment in simulated intestinal fluid at pH = 6.8 [7]. For the P-1 test, this point is reached after 4 h for M-HCl (83.25%) and 9 h for HNK (93.26%), with a maximum release of 99.27% (M-HCl) and 98.66% (HNK) at the end of the test, and for the P-2, we have released 83.04% (M-HCl) after 7 h, 90.76% (HNK) after 9 h with a maximum 98.20% (M-HCl), 98.83% (HNK) after 12 h (Figure 1). The statistical analysis of the in vitro release test data revealed that there were statistically significant differences in the release profile of M-HCL between P-1 and P-2 (*p* = 0.041). Conversely, no statistically significant differences were observed in the release profile of HNK between the two analyzed samples (*p* = 0.938). The two analyzed samples are in line and fit within the release profile for prolonged-release dosage forms, according to “5.17. Recommendations on methods for dosage forms testing—5.17.1 Recommendations on dissolution testing” and “2.9.3. Dissolution test for solid dosage forms” and from European Pharmacopoeia, 11th edition [7].

The obtained results confirm the efficacy of hydrophilic polymers, such as Carbopol^®^ 71G NF and Noveon^®^ AA-1, in controlling the prolonged release of active substances from matrix tablets. These observations are consistent with literature data, which have demonstrated that such polymers form swellable matrices that allow controlled release through diffusion and swelling mechanisms [23,24,25]. Carbopol matrices dissolve less readily at lower pH values, which is advantageous for oral therapy as it minimizes the loss of the active ingredient in the stomach—an aspect particularly relevant to the polyphenol category, to which HNK also belongs [24].

### 3.2. Theoretical Design

The ground of applying fractal/multifractal analysis for the drug release process represents the assumption that the motion of release drug particles takes place on fractured lines as a result of the collisions with other drug particles, polymeric matrix and release environment [26,27,28]. The fractured lines are continuous, but non-differentiable curves, named fractal/multifractal curves. Further, Nottale’s Scale Relativity Theory [29] and/or Extended Scale Relativity Theory were developed and applied to different types of drug release systems [30,31].

In such a context, let it be considered that the dynamics of controlled drug release from the previously mentioned system can be described, according to [30,31], by the fractal equation:

(4) $$\partial_{t}Q+Q\;\partial_{X}Q+\Lambda {\left(dt\right)}^{\left(\frac{3}{D_{F}}\right)-1}\partial_{X}^{3}Q=0$$

where

$$\partial_{t}=\frac{d}{dt},\;\partial_{X}=\frac{d}{dX},\;\partial_{X}^{3}=\frac{d}{dX^{3}}$$

In the previous equation, X defines the spatial coordinate, depicted by continuous and nondifferentiable mathematical functions which rely on the scale resolution. The temporal coordinate, *t*, is depicted by continuous and differentiable mathematical functions which do not rely on scale resolution. dt defines the scale resolution. Q can be linked with the released drug mass, defined in percentages. Λ characterizes the differentiable-nondifferentiable scale transition coupled with the forward and backward drug release dynamics, respectively. $D_{F}$ represents the fractal dimension [32] of the motion curves of the structural units of any drug release system. 

By following the methodology presented in [19,20], the general solution of (4) can be found in the form of the expression:

(5) $$Q\left(X,\;t\right)=Q_{0}+a\;cn^{2}\left(\frac{X-V_{0}t}{\Delta };s\right)$$

with

(6) $$Q_{0}=\frac{a}{s^{2}}\left[1-s^{2}-\frac{E\left(s\right)}{K\left(s\right)}\right]\;,\;\;\Delta =\frac{\langle X\rangle }{2K\left(s\right)}$$

In Equations (5) and (6), a is an amplitude, *V*_0_ is a phase speed, K(s) and E(s) are the complete elliptic integral of the first and the second kind, respectively, $\langle X\rangle$ is the characteristic length of DCR system, and *cn* denotes the Jacobi elliptic function of *s* modulus [33].

It must be noted that, since the parameters of the releasing process (a, *V*_0_, $\langle X\rangle$, and *s*) depend on $\Lambda {\left(dt\right)}^{\left(\frac{3}{D_{F}}\right)-1}$, the dynamic of DCR is achieved through fractal cnoidal oscillation modes of the complex system (support/drug) [34]. For *X* = 0, the drug mass released in time from the support is given by means of integral

(7) $$M\left(t\right)=\int Q\left(0,\;t\right)dt=\int Q_{0}dt+a\int cn^{2}\left(\frac{-V_{0}t}{\Delta },s\right)dt$$

From here, with the non-dimensional variables,

(8) $$\tau =\frac{t}{t_{0}},\;\;\;\;\;\;\;\;\;\;\theta \left(\tau \right)=\frac{M\left(t\right)}{a\cdot t_{0}},\;\;\;\;\;\;\;\;\;\;t_{0}=\frac{\langle X\rangle }{2V_{0}}$$

this results in

(9) $$\theta \left(\tau \right)=\left[\frac{1}{s^{2}}-1-\frac{E\left[s\right]}{s^{2}K\left[s\right]}\right]\tau +\frac{E\left\{Am\left[K\left[s\right]\tau ;s\right];s\right\}\left\{-1+\frac{1}{s^{2}}{cn}^{2}\left[K\left(s\right)\tau ;s\right]\right\}+\left(1-\frac{1}{s^{2}}\right)K\left(s\right)\tau }{K\left(s\right)dn\left[K\left(s\right)\tau ;s\right]{\left\{1-s^{2}{sn}^{2}\left[K\left(s\right)\tau ;s\right]\right\}}^{\frac{1}{2}}}$$

where E(Am), dn and cn are the standard Jacobi elliptic functions of *s* modulus [33].

**Figure 2:**
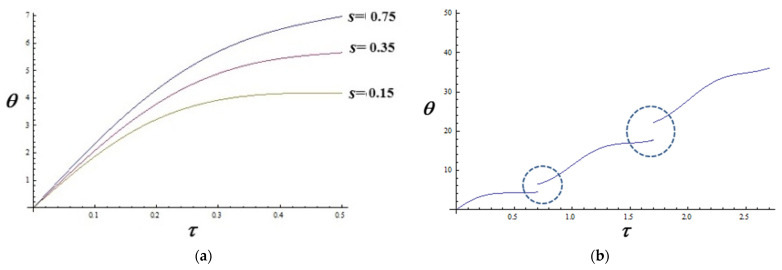
(**a**) The dependence of the drug released vs. time τ for various values of *s* parameters, at low time-sequences; (**b**) behaviors associated with fractional drug release effects.

Assuming further that to the *s* modulus of the elliptic functions from (9) one can associate only the scale resolution, shown in Figure 2a, using the non-dimensional variables (7), the dependence of the drug released mass both on the time τ and scale resolution s, while in Figure 2b, such time-dependences are detailed for various values of *s* parameters. 

(i) At relatively short time sequences and at given scale resolutions, the process of drug releasing mimics standard behaviors which are specified through classical and empirical laws of the releasing kinetics (Figure 2a); (ii) for relatively high time-sequences and assuming the functionality of a scale superposition principle [34], the drug releasing process can mimic behaviors associated with fractional drug release effects (Figure 2b). However, such behaviors imply both special topologies and strange mechanisms of the type of composed fermions, etc. [35,36].

Therefore, the proposed model can describe the previously shown experimental data, presented in Figure 1a,b.

## 4. Conclusions

This study successfully developed and characterized extended-release matrix tablets containing metformin hydrochloride (M-HCl) and honokiol (HNK), utilizing hydrophilic polymers to achieve controlled release profiles. Results from the in vitro dissolution tests demonstrated that the release behavior of both drugs is highly dependent on polymer concentration, with Carbopol^®^ 71G NF and Noveon^®^ AA-1 playing a critical role in modulating the release kinetics. The findings showed that M-HCl achieved 80% release within 4 to 7 h, while HNK exhibited a slower release, reaching the same threshold between 9 and 10 h. This differential release pattern is advantageous for optimizing antidiabetic treatment, as it enables tailored therapeutic windows that align with the physiological needs of patients over extended periods. The mathematical modeling employed in this study provided a deeper understanding of the drug release mechanisms. The application of fractal and multifractal dynamics allowed for accurate predictions of release profiles, demonstrating that while classical kinetic models are useful at early time intervals, they do not fully capture the complexity of extended-release systems. These advanced models confirmed that, at longer time intervals, the drug release process is governed by multifractal behaviors, indicating the involvement of non-linear diffusion and erosion processes within the polymer matrix. Moreover, this study underscores the importance of polymer concentration in determining the rate and extent of drug release. The hydrophilic polymers used in these formulations effectively formed gel-like matrices that controlled drug diffusion and minimized the risk of dose dumping, a critical factor for maintaining therapeutic efficacy and patient safety. In conclusion, the combination of metformin hydrochloride and honokiol in extended-release matrix tablets presents a promising strategy for enhancing the treatment of type 2 diabetes. The controlled release properties achieved with the selected polymers not only improve the pharmacokinetic profiles of both drugs but also enhance patient adherence by reducing dosing frequency. This research also lays the groundwork for further exploration into the application of multifractal modeling in drug delivery systems, providing a robust framework for future formulation development and optimization.

## Data Availability

The original contributions presented in the study are included in the article, further inquiries can be directed to the corresponding author.

## Abbreviations

DAD	Diode Array Detector
HNK	Honokiol
HPLC	High-Performance Liquid Chromatography
M-HCl	Metformin Hydrochloride
US	United States of America
